# Reversible and Irreversible Color Change during Photo and Thermal Degradation of PolyphenyleneSulfide Composite

**DOI:** 10.3390/polym11101579

**Published:** 2019-09-27

**Authors:** Victor B. Ivanov, Vladimir V. Bitt, Elena V. Solina, Alexander V. Samoryadov

**Affiliations:** 1 Semenov Institute of Chemical Physics of Russian Academy of Sciences, 4 Kosygin Street, Moscow 119991, Russia; lab.evi@rambler.ru; 2 Research & Production Company “Polyplastic”, 14 General Dorokhov Street, Moscow 119530, Russia; bitt@polyplastic.ru; 3 Interdepartmental Center for Analytical Research in Physics, Chemistry, and Biology, Presidium RAS, 65 Profsoyuznaya Street, Moscow 117342, Russia; a2612sam@yandex.ru

**Keywords:** photodegradation, polyphenylene sulfide, reversible color change, thermodegradation

## Abstract

The effect of the light spectral composition and temperature on the change of color characteristics and reflection spectra during the irradiation of polyphenylene sulfide reinforced by short glass fibers in the SUNTEST apparatus was analyzed. The scales of reversible color change upon successive exposure to total radiation corresponding to the sunlight spectrum and to visible light wereevaluated and the possible mechanisms for the observed effects are discussed. The features of the color change upon visible light irradiation of previously thermally aged samples wereconsidered. Possible causes for deviation from the Arrhenius law during thermal aging of the composite are discussed. It wasdemonstrated that even with a significant change in color, the physicomechanical and electrotechnical characteristics of the composite only changedslightly or remained virtually at the same high level.

## 1. Introduction

Polyphenylene sulfide (PPS) possesses a number of important properties. First, of all, its high temperature and heat resistance; chemical resistance to acids, alkalis, and organic solvents; crack resistance; low water absorption; low creep; and excellent electrical characteristics should be noted [1,2]. Due to these features, materials based on PPS are widely used in electrical and electronics as well as in other industries [3,4,5].

The disadvantage of PPS is its relatively low light resistance, which especially manifests in the rapid change in color [6,7,8]. Coloring in photo and thermal degradation processes is a specific property of the PPS macromolecules themselves and is manifested for both the linear and crosslinked polymer. The color change of the irradiated samples is almost independent of the molecular weight and origin of the end groups [6]. Noticeable coloring is also observed during the thermal degradation and thermal oxidation of PPS [8]. Conventional light stabilizers in PPS are not sufficientlyeffective [7,9]. It can be assumed that the low efficiency of antioxidants in the photo and thermal destruction of PPS at low temperatures is not due to the freeradicals, but to the intramolecular origin of the process with the formation of polyconjugated structures that absorb in the visible and long-wave UV spectral regions. This, in turn, may lead to a significant sensitivity of samples both to the short-wave radiation absorbed directly by the –C_6_H_4_–S– units and to the long-wave UV and visible light. Therefore, to increase the light resistance of PPS-based materials, very high concentrations of UV-absorbers are required [7]. Another proposition is using coatings of different polymers, for example, polydopamine [10]. Expansion of the spheres of PPS use, particularly in aerospace engineering, has stimulated investigations of the effects of the external factors on changes in the properties of composite materials based on PPS [11,12,13,14,15,16,17,18,19,20,21,22,23,24,25,26,27]. Glass and carbon fibers [11,12,13,14,15,16,17] and nanoparticles [18,19,20,21,22,23,24] are commonly used as active fillers. Materials based on mixtures of PPS with other polymers [25,27] are of particular interest.

When analyzing the aging processes of materials under natural conditions, it is nearly always necessary to account for the influencing factors that change with time due to daily and seasonal fluctuations and other causes. It has beenshown that even in the relatively simple case of the physical aging of PPS under non-isothermal conditions, temperature fluctuations can bring about a number of specific qualities [28]. In this particular case, the quantitative description of the material aging due to the use ofwell-known models developed for polymers of other classes can be designed. However, for more complex cases, for instance, during aging under conditions of varying spectral compositions of light, or with the alteration of thermal and photochemical effects, no universal models exist. Therefore, as has been pointed out in numerous occasions in the literature, each kind of material, especially those practically important and promising, needs to be investigated separately.

The main goal of this work was to analyze the effect of light and heat, in particular, their sequential effect as well as the sequential effect of light of different spectral composition on the color change kinetics of a composite material based on glass-filled PPS designed for wide usage.

The applied goal of the study was to assess the possibility of using colorimetry for the analysis of photo and thermal degradation of composites based on PPS.

The results of preliminary studies presented in the patent [29] and in the patents and papers cited therein, indicate that for the purpose of producing materials with high physical, mechanical, and electrical characteristics based on glass-filled PPS, it is advisable to use a polymer of linear or crosslinked structure with melt flow rates of 50 to 800 g/10 min (at 310 °C), and as a filler (i.e., glass fiber with a diameter of 5 to 15 microns). These indicators provide technological processing of the composition by the methods of extrusion or injection molding. It wasfound that the composite containing 40 wt.% of glass fiber possesses the maximum or closely spaced characteristics. A composite containing 25 or 55 wt.% of glass fiber has a significantly lower tensile strength (by 29.2 and 7.8%, respectively) and impact strength (by 41.4 and 16.6%, respectively). Moreover, a composite containing 25% of the filler has a significantly lower tensile modulus (by 34.6%), flexural stress at maximum load (by 24.2%), and flexural modulus (by 60.6%) [29]. Therefore, it the composite with the glass fiber content of 40 wt.% was selected as the object for study.

## 2. Materials and Methods

A linear PPS powder with a melt flow rate of 250 g/10 min (316 °C, 5 kg) and a melting point of 278 °C grade 1330C (NHU-PPS Polymer, Shaoxing, Zhejiang, China) was used in the work. Chopped fiberglass of the grade 910A-10P 45 mm (ADV) with a diameter of 10 μm was used as a filler. To increase the thermal stability, the composition contained a mixture of bis(2,4-di-tert-buthylphenyl)pentaerithritol phosphate (0.3 wt.%) and N,N′-hexamethylene-bis [3-(3,5-di-tert-buthyl-4–hydroxyphenyl- propionamaid)] (0.3 wt.%).

Samples of the composite in the form of granules ~2–3 mm in size were obtained by mixing powdered PPS, thermal stabilizers, and fiberglass in a twin-screw extruder at a temperature of 310 °C and a conveyor screw rotation speed of 50 rpm.

Test samples represented bydisks of 2 mm thick and 50 mm in diameter as well as with multipurpose samples of type A1 according to ISO 20753:2008 wereinjection molded from glass-filled PPS under the following conditions: Casting temperature of 320 °C; casting pressure of 100 MPa; molding pressure of 75 MPa; plasticization pressure of 7 MPa; the mold temperature of 140 °C; the holding time under pressure of about 15 to 20 s; and a holding time under cooling of about 20 to 25 s.

Thermal aging was performed at temperaturesof 90, 100, 110, and 120 °C under conditions of forced air supply in a low-temperature furnace SNOL-3.9.3.9.3.6/3.5-2N (Gomel, Republic of Belarus).

Photochemical aging was applied using the SUNTEST XLS + (Atlas, Linsengericht, Germany) or Q-SUN (Q-Lab, Westlake, OH, USA) device utilizing filter systems that matched the spectral composition of the sunlight under natural conditions (radiation wavelength greater than 290 nm), with a light intensity of 500 W/m^2^ (SUNTEST XLS +) or 1000 W/m^2^ (Q-SUN). The samples were continuously irradiated from1 to 120 h. To estimate the activation energy of the photochemical destruction of PPS, the SUNTEST tests were carried out at two valuesof a black panel temperature of 50 °C or 60 °C.

The determination of the color characteristics and reflection spectra in a wavelength range of 400–700 nmwas implemented using a ColorFlex spectrophotometer (Hunter Lab, Reston, VA, USA) under the following mode: 45/0°, observation angle 10°, and light source D65. Used as the main criterion, the value of the color difference, ΔE, in the CIELAB-76 [30] system was determined by Equation (1):

ΔE = [(ΔL*)^2^ + (Δa*)^2^ + (Δb*)^2^]^1/2^, (1)

where ∆L* = L*_0_−L*_i_, ∆a* = a*_0_−a*_i_, ∆b* = b*_0_ −b*_i_; in this case, the index ‘0′ refers to the sample before the test, and the index ‘i’ refers to the sample after a certain period of testing. The values of L*, a*, and b* were determined, resulting directly from the measurements using the standard procedure for processing reflection spectra by the ColorFlex instrument software.

IR spectra were recorded using a Nicolet 6700 Fourier spectrometer (Thermo Fisher Scientific, Madison, WI, USA) with an iTR ATR (Thermo Scientific, Madison, WI, USA) attachment (ZnSe) in a range of 400–4000 cm^−1^ in the accumulation mode (1024 scans) at a resolution of 2 cm^−1^. The data were processed and analyzed using the OMNIC software.

The relaxation properties and phase transition temperatures were studied by the dynamic thermomechanical method using a DMA ARES2000 device (TA Instruments, New Castle, DE, USA) in shear mode, where a sinusoidal stress of 0.03% of the specimen material strength with the frequency 1 Hz was applied to the sample (i.e., stress that did not lead to any change in the material analyzed). The rate of temperature rise from −50 °C to 250 °C was 5 °C/min.

Thermogravimetric analysis of the composite was performed on a METTLER TGA/SDTA 851e instrument (Mettler Toledo, Zaventem, Belgium) in air at the following conditions:

- The weight of the test sample, 10–15 mg;
- The temperature range covered, 30–800 °C;
- The heating rate 2, 5 or 10 °C/min; and
- The gas flow rate, 80 mL/min.

The modified Kissinger (Kissinger-Akahira-Sunose, KAS) method [31] was used to determine the activation energy of thermodegradation (Equation (2)):

ln(β_i_/T_αi_) = const− E_α_/(RT_α,i_), (2)

where β_i_ is the heating rate at the i-th temperature program; T_α,i_ is the temperature at which a degree of conversion α is attained at a given value β_i_; E_α_ is the activation energy of the process at a degree of conversion α; and R is the universal gas constant.

The tensile and flexural strength and tensile and flexural modulus of elasticity were measured with an Instron 1185 universal device (Nordwood, MA, USA). Tensile strength was determined at the rate of 5 mm/min, and the modulus of elasticity at the rate of 0.5 mm/min in the range of 0.05–0.25%. Flexural strength at break and flexural modulus of elasticity (three point method) were determined at the rate of 0.5 mm/min. The Charpyunnotched impact strength was measured on an impact testing machine Zwik HIT5.5. PC (CEAST, Genova, Italy) at 23 °C. To ensure the reliability and reproducibility of the results obtained, 10 samples were tested in determining each physicomechanical parameter.

Volume and surface resistivities were determined on five samples of each species using an EG-13A teraohmmeter (Smolensk, Russia).

## 3. Results and Discussion

**Figure 1:**
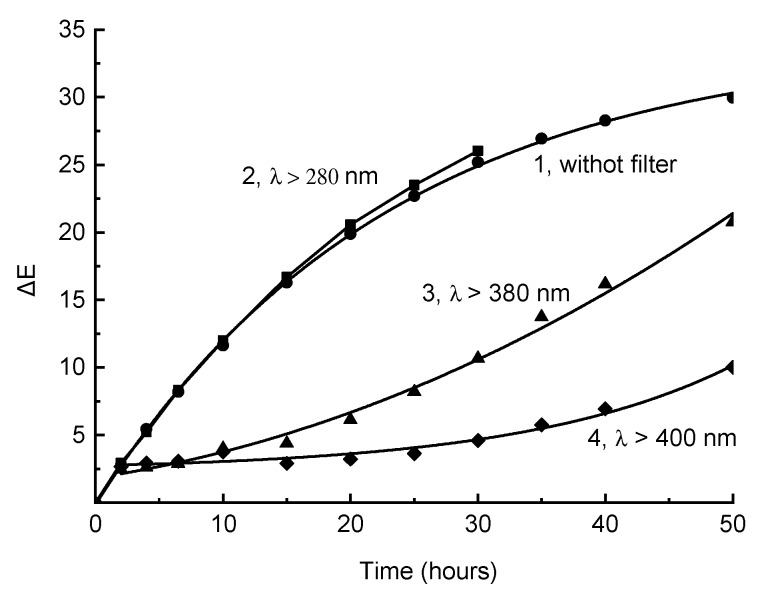
Kinetic curves of the changes in color difference during irradiation of the composite in the SUNTEST device without supplementary filters (1) and with light filters cutting off the shortwave radiation of λ < 280 nm (2), λ < 380 nm (3), or λ < 400 nm (4). The black panel temperature was 60 °C.

Irradiation of the disc-shaped samples in the SUNTEST apparatus with light simulating solar radiation with no additional light filters led to a significant change in color (Figure 1, curve 1), which is quantitatively recorded by the color difference value, ΔE. At deeper stages, the initial rapid increase in ΔE slowed down gradually. Changes in the spectral composition of incident light on the samples, using light filters that cut off short-wave radiation (BS8, λ > 380 nm or ZhS11, λ > 400 nm) significantly affect the kinetics of the process (Figure 1, curves 3 and 4). In this case, noticeable induction periods were observed, which were especially well expressed when exposed to light with λ > 400 nm. It is of importance that when the induction period was complete, the color difference under irradiation through the BS8 light filter (λ> 380 nm) occurred at almost the same rate observed when the sample was irradiated without additional light filters, despite a decrease in the overall intensity of incident light on the sample, and a decrease in the shortwave UV light component. Note that the effect could not be attributed to the known effect of additional “under glass” sample heating, because when using a BS4 optical filter (λ> 280 nm), which does not affect the spectral composition of the SUNTEST light, the process kinetics remained almost unchanged (Figure 1, curve 2).

**Figure 2:**
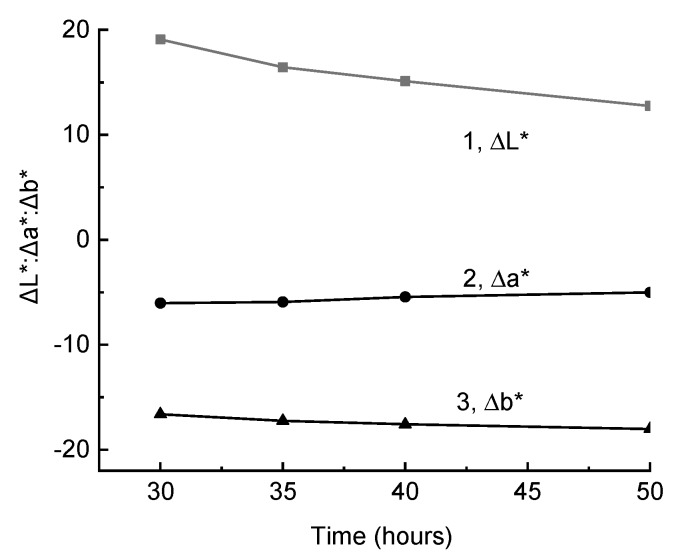
The change in color coordinates ΔL* (1), Δa* (2), and Δb* (3), when irradiated by light of λ > 400 nm (ZhS11 light filter) of a composite sample that was previously irradiated by the total light during 30 h.

Gradual reaching of the ΔE value of the quasistationary level may be associated with the dual function of light: At the same time, it initiatesthe formation of polyconjugated structures responsible for the composite coloring, and destroysthese structures directly or with the intermediate participation of free radicals that initiate oxidation. This suggests that, upon irradiation of a sample that was previously colored by total radiation by visible light, a change in color should be observed, and rightly so, such an effect was observed (Figure 2). The fact that L* changed to the greatest extent is important and interesting. The increase in L* led to a decrease in ∆L* when compared with the sample after irradiation by total light (Figure 2, curve 1). To a lesser extent, a* changed (Figure 2, curve 2, a decrease in the absolute value of Δa*). Changes in L* and a* were, to some extent, compensated by an increase in b*, which, with a large absolute value of Δb*, led to significantly less change in color difference after 20 h of exposure to visible light (ΔE = 3.39) than in lightness (ΔL* = 6.35).

**Figure 3:**
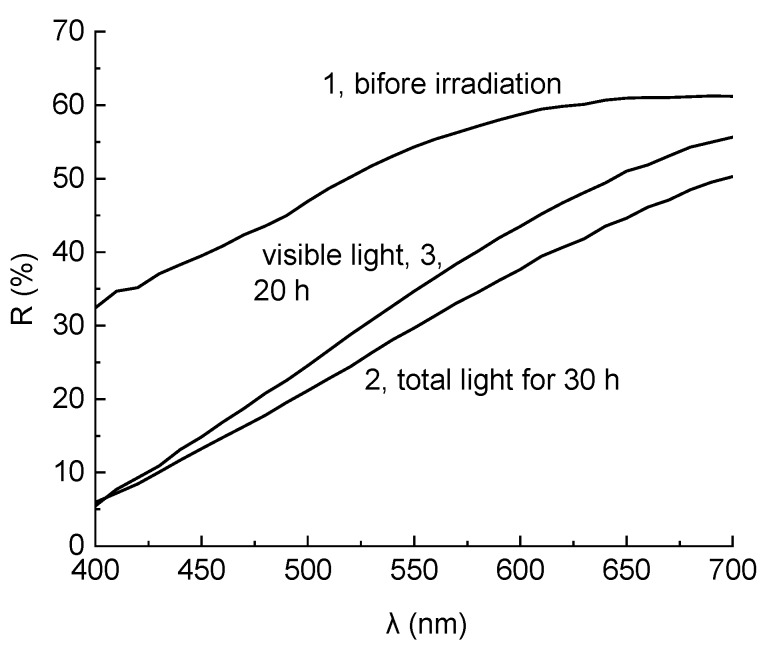
The reflection spectra of the composite before irradiation (1) and after irradiation by total light for 30 h (2), and additional irradiation by light of λ > 410 nm (ZhS12 light filter) for 20 h.

The corresponding effects can also be estimated by comparing the reflection spectra of the samples (Figure 3). It is apparent that the irradiation by total light in the SUNTEST device brings about a decrease in the reflection coefficients in the entire visible region. However, the scale of effect increased with the decrease in the wavelength, and reached its maximum values at 400–450 nm, which is expressed by yellowing of the sample. On the contrary, irradiation by visible light using supplementary ZhS11 light filter led to a more significant increase in reflection in the long-wave region, while if the irradiation was close to 400 nm, virtually no changes were observed. This, of course, led to an increase in lightness while maintaining, and even increasing to a lower extent, the degree of yellowness assessed by the value of b*.

**Figure 4:**
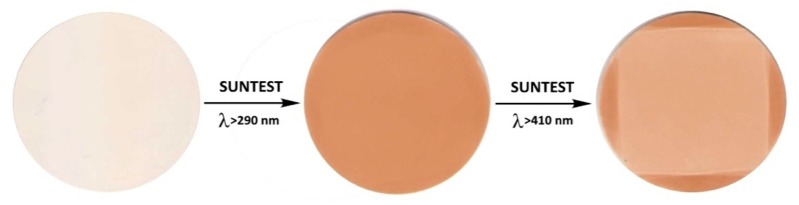
Samples of the composite before testing (**left**) and after irradiation with full light for 30 h (**middle**) or subsequent irradiation with visible light for 20 h (**right**).

Note that the observed effect waspractically significant, since the change in color, when the spectral composition of light varies, couldbe clearly seen with an unaided eye (Figure 4), even at short times of irradiation.

**Figure 5:**
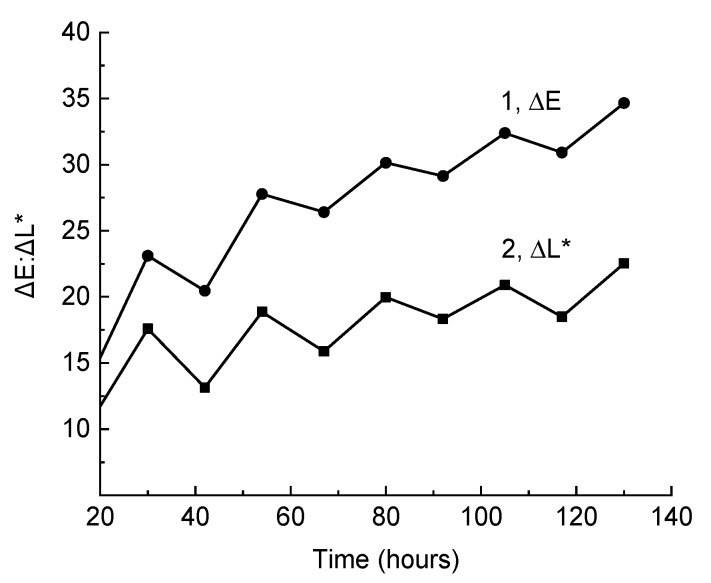
Change in the color difference, ΔE (1), and lightness, ΔL* (2), with periodic irradiation of the composite by total or filtered light (λ > 400 nm, ZhS11 light filter). The sample was pre-irradiated by total light for 30 h (the firstpoint on the broken line).

Processes of coloring for the composite under the action of total radiation in the SUNTEST device and partial bleaching caused by visible light couldbe repeated many times (Figure 5). At the same time, both in the first and in subsequent cycles, the main contribution to the change in color difference, ΔE, wasmade by the change in lightness L*. Thus, the composite, to some extent, possesses photochromic properties. This characteristic property of the PPS photodegradation must be considered in the course of photostability testing and prevent the action of the scattered daylight or the light of luminescent lamps during interruptions of the test process.

**Figure 6:**
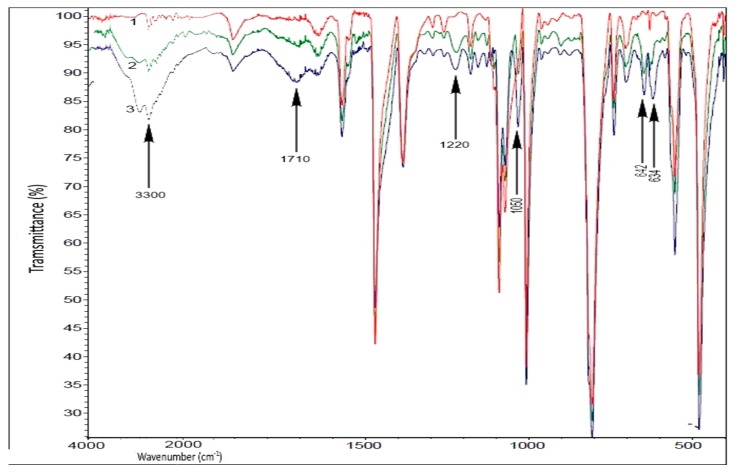
The Fourier Transform Infrared Spectra FTIR spectra of the composite surface before irradiation (1) and after irradiation by total light in the SUNTEST device for 30 h (2) and additional irradiation by light of λ > 400 nm for 20 h. The arrows indicate the bands arising under the influence and full/or visible light at 634 (–C_6_H_4_–S(=O)_2_–), 642 (–S(=O)_2_–C_6_H_4_–S(O_2_)–), 1050 and 1220 (C–O), 1710 (C=O), and 3300 cm^−1^ (O–H groups).

In accordance with the hypothesis formulated above on the dual effect of light on PPS coloring, partial discoloration caused by visible light was due to the oxidation of polyconjugated structures. The comparison of IR spectra indicates that the most noticeable changes after irradiation by both primarily total light (Figure 6, spectrum 2) and subsequent irradiation by visible light (Figure 6, spectrum 3) wereobserved at 1050 and 1220 cm^−1^ (C–O groups), a broad band with the maximum at 1710 cm^−1^ (C=O groups), and 2600–3690 cm^−1^ (О–Н groups). Oxidation also affects the S atoms with the formation of sulfogroups (absorption bands at 634 and 642 cm^−1^ of the groups –C_6_H_4_–S–C_6_H_4_–S(=O)_2_– and –S(=O)_2_–C_6_H_4_–S(=O)_2_–). It is of interest that subsequent irradiation with visible light led to a significant increase in the band at 642 cm^−1^ and, as a consequence, a change in the ratio of the band at 634 cm^−1^ and 642 cm^−1^. This may be due to the local occurrence of the process: In areas where polyconjugated structures have already been formed and there are single groups –S(=O)_2_–, the oxidation of polyconjugated structures and the oxidation of neighboring sulfide groups occurred under the influence of visible light. The fact that the band intensities in the absorption region of O–H groups was higher than in the absorption region of C=O groups is of interest. Since extinction coefficients of carbonyl and, especially, carboxyl groups weresignificantly greater than that of the hydroxyl groups, this result unambiguously indicates the relatively low concentrations of carboxyl groups on the irradiated surface, and hence, a relatively small number of macromolecular chain scissions. A significant increase in these bands upon irradiation by visible light indicates an intense oxidation of the composite surface. The formation of such functional groups led to a qualitative change of the ability to be wetted come moist with water. In this case, a drop of water applied on the composite spread over the surface, in contrast with the non-wettable initial composite.

Photochemical coloring increased with temperature. Unlike a number of other engineering thermoplastics [32], the kinetic curves cannot be superimposed on each other through transformation along the time axis. This actually means that the activation energy depends on the degree of coloring (conversion degree). A similar phenomenon of the activation energy dependence on the conversion degree, in particular, is well known for the thermal decomposition processes studied by thermogravimetric analysis [33,34,35]. Based on the natural assumption that for photodegradation of the composite as well as the photo and thermal degradation of polymeric materials considered as examples in [32], the Arrhenius law is satisfied, and the activation energy E_α_ at the conversion degree (degree of coloring), α, can be estimated by Equation (3):

E_α_ = R[(T_1_T_2_)/(T_2_ − T_1_)]lnk_α_, (3)

where T_2_ and T_1_ are the temperatures at which photodegradation occurs; k_α_ = t_2_/t_1_ is the acceleration factor; t_2_ and t_1_ are the times at which a certain degree of conversion, α (a certain value of the color difference ΔE), is reached at temperatures T_2_ and T_1_; and R is the gas constant. The values of activation energies thus obtained are summarized in Table 1.

It can be seen that as the color difference increased during irradiation, the activation energy decreased first, reaching a minimum of about 17 kJ/mol, and then increased gradually to 36–37 kJ/mol. This feature is apparently due to a change in the ratio of the formation and consumption of polyconjugated structures at different stages of the process. At the initial stages, the formation of precursors and the polyconjugated structures themselves absorbing in the visible region is the limiting factor. At deep stages, due to the increase in the consumption rate of polyconjugated structures, the rates of their formation and consumption approach one another, and their content gradually reaches a quasistationary level. The middle section of the kinetic curve of ΔE change represents a transition region between these modes. In general, the average activation energy of photodegradation of the studied composite wasin the same sequence as the activation energy of other engineering thermoplastics (7–28 kJ/mol) [32].

Due to the high absorption of PPS and products of its thermal and photodegradation, first of all, as their polyconjugated structures are formed during processing and subsequent exposure, the photodegradation process takes place in a thin (20–40 μm thick, depending on the conditions and duration of exposure) surface layer. With the mechanical removal of this layer, the color of the sample wasalmost completely restored.

Therefore, the mechanical and electrical characteristics changed slightly, even with long-term exposure (Table 2).

Note that the failure of the irradiated samples as well as the initial ones wasbrittle and occurred at small strain values of 1.9% to 2.0%, as typical for the filled polymeric materials.

**Figure 7:**
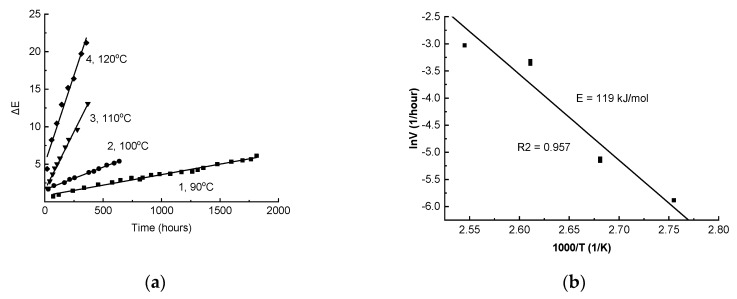
(**a**) The increase in color difference, ΔE, during the isothermal aging of the composite in air at 90 °C (1), 100 °C (2), 110 °C (3), or 120 °C (4); (**b**) The dependence of the stationary rate of the increase in color difference, ΔE, during the thermal aging of the composite on the temperature in the Arrhenius equation coordinates.

Coloring was also observed during the thermal aging of the composite (Figure 7a). This process rate was noticeably less than for photochemical aging. In particular, a noticeable change (ΔE ~ 2) at 90 °C occurred only after 1000 h exposure. It should be noted that at the initial stage of aging, the change in ΔE always occurs more quickly (a “jump” of ΔE was observed). The higher the temperature, the greater the “jump” magnitude (Figure 7a). The effect is apparently associated with the presence of polyconjugated structure precursors (polyconjugated structures with a small conjugation length, which do not absorb in the visible region) in the sample. These precursors, as well as the polyconjugated structures that color the material, can be formed during the thermal degradation of PPS during the obtaining of the samples, which wascarried out at very high temperatures. At the later stage, the change in ΔE occurred at almost a constant rate over a long period (Figure 7a).

**Figure 8:**
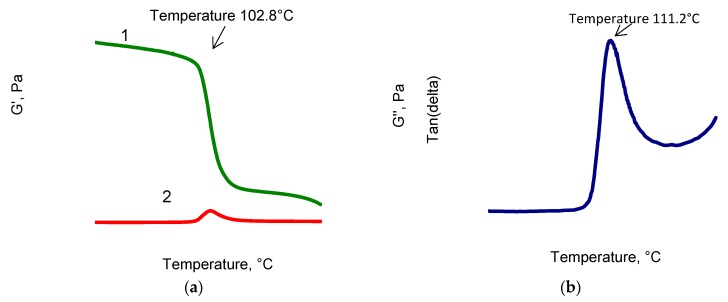
(**a**) Dependences of the shear modulus of (1) and elastic modulus G^II^ loss (2); (**b**) Dependence of the mechanical dissipation factor on temperature.

The dependence of the stationary rate of increase in color difference, V, on temperature in the range of 90–120 °C is described by the Arrhenius equation, but the deviations of the experimental points from the straight line in lnV-1/T coordinates werequite large (R^2^ = 0.957, Figure 7a). It is apparent that in the range of 100–110 °C, there was a more significant increase in the rate than could be expected on the basis of the extrapolation of data at 90 °C and 100 °C. The kinetic dependences of the ΔE changes at 100 °C and 110 °C werewell reproduced. Therefore, in two independent experiments at 100 °C, the speed values were 5.75 × 10^−3^ and 6.0 × 10^−3^ 1/hour, and at 110 °C were 3.45 × 10^−2^ and 3.61 × 10^−2^ 1/h (the merging points in Figure 7b). Apparently, the acceleration at 110 °C is associated with a sharp change in the molecular mobility of PPS when passing over the glass transition point, clearly recorded by the dynamic thermomechanical analysis (Figure 8). 

As can be seen from the data of the dynamic thermomechanical analysis (DTMA) of the PPS samples, the shear modulus (Figure 8a, curve 1) of the sample remained unchanged up to the temperature of ~90 °C and, with a further increase in temperature, decreased in the region of 100 °C to 150 °C and then to 250 °C. Such behavior of the modulus was due to the PPS glass transition as follows from the data of the elastic modulus G^II^ losses (Figure 8a, curve 2) and the mechanical dissipation factor (Figure 8b).

The activation energy of the thermal aging of the composite determined by a change in the coloring rate with a temperature increase from 90 °C to 100 °C was 110 kJ/mol. This value hadgood agreement with the results obtained in the process of studying the thermally induced luminescence of a PPS/elastomer mixture (113 ± 25 kJ/mol) [25]. The activation energy of the thermal aging of the composite estimated according to Figure 7 in the entire studied range of 90–120 °C was noticeably higher (119 ± 17 kJ/mol). The closely spaced value (129 ± 17 kJ/mol) was determined for the composite by the TGA integral isoconversional method (KAS) at lower conversion degrees (1–2%).

Unlike photodegradation, thermal degradation propagates into layers of the composite that are substantially located more deeply from the surface. With the mechanical removal of а layer up to 100 μm thick, the color of the samples changed slightly. Visual assessment indicated a significant coloring in the middle of the studied samples, that is, at a depth of about 1 mm.

As shown above, when the test conditions for light stability werechanged by varying the spectral distribution of light, the following qualitative effects wereobserved: Drastic change in the shape of kinetic curves and even reversible color change. In the case of thermal degradation, such qualitative effects wereabsent. A sharp increase or decrease of the test temperature didnot lead to unexpected effects: The coloring proceeded at a rate typical of this new temperature mode. In this respect, PPS is different from polyvinyl chloride, which is also strongly colored at elevated temperatures, but for which both the enhancement and weakening of color with the change of aging temperature were observed [34,35].

The coefficient k_α_ can be estimated using Equation (3) and by loweringthe limit of the activation energy value to 102 kJ/mol, when the temperature changes from 90 °C to 60 °C, consequently, the rate of coloring due to thermodegradation (the change in color difference) under conditions of photostability testing can be estimated. At 60 °C, the rate value of thermodegradation will be 6.93 × 10^−5^ 1/hour. Therefore, achieving the value of ΔE = 2 (the minimum reliably fixed visual level) will require 2.9 × 10^4^ h, or about three years. At 50 °C, to achieve the same color change, about 10 years is required. These estimates, as well as the values of the rates at 90 to 100 °C, indicate that the contribution of thermal aging itself in the study of PPS composite photodegradation can be neglected. The obtained data and estimates based on them indicate a very high resistance of the material to thermal aging.

**Figure 9:**
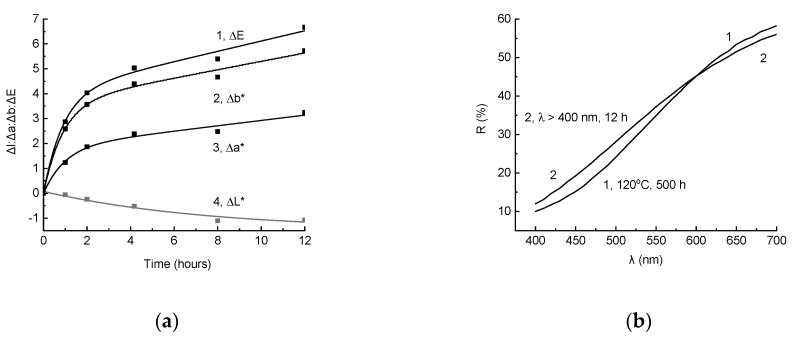
(**a**) Change in color difference ΔE (1) and color coordinates Δb* (2), Δa* (2), and ΔL* (4) when irradiated by visible light (λ > 400 nm, light filter ZhS11) of the composite sample preheated at 120 °C for 500 h. (**b**) The reflection spectrum of the composite sample after heating at 120 °C for 500 h (1) and then irradiated by light of λ > 400 nm (light filter ZhS11) for 12 h (2).

It was found, however, that when irradiating the previously thermally aged samples by visible light, their color changed noticeably (Figure 9a). In this case, the difference from a similar phenomenon of color change during photodegradation with varying exposure conditions wasthat the decrease in ΔE was generally determined by Δb* (Figure 9a, curves 1 and 2), rather than by ΔL*, as it appears during photodegradation. This is due to the peculiarities of changes in the reflection spectra during the irradiation of thermally aged samples (Figure 9b). It is apparent that in the region of λ < 600 nm the reflection coefficient increased, and, in contrast, in the region of λ > 600 nm, it decreased. Therefore, in this case, the color change seemed to bevisually less pronounced than when the preliminarily photochemically aged samples were irradiated by long-wave light.

Note that the “bleaching” effect of visible light on previously thermally aged samples makes it possible to explain, in a natural way, the phenomenon of the sharp initial change in the color difference that occurred when samples were irradiated by filtered light (Figure 1). The studied samples were obtained by a routine industrial method of injection molding, which was carried out at high temperatures. As a result, they were colored due to the thermal degradation of PPS (mean values L* = 78 ± 1; a* = 1.4 ± 0.2; b* = 14.5 ± 0.7). Therefore, irradiation by long-wave light absorbed predominantly by polyconjugated structures, leads to their consumption, and consequently, to a decrease in yellowness (decrease in b*). Thus, for example, after 2 h of irradiation by light at λ > 400 nm (Figure 1, curve 4), L* = 77.20; a* = 1.07; b* = 12.46; with initial values L* = 78.59; a* = 1.26; b* = 14.73. At this initial stage, the change in b* (Δb* = 2.27) makes the main contribution to the change in color difference (ΔE = 2.67). It is significant, however, that in this case, weakening rather than deepening of the original color happens. At deeper stages, despite a significant increase in b* (up to 16.68 within 30 h), the main contribution to the color difference is made by a decrease in L* (30 h after, ΔL* = 4.14; ΔЕ = 4.60). Since a sharp change in color difference at the beginning of irradiation is more pronounced, the more significant coloring is a result of thermal aging when a sample or product is obtained, as thermal stabilizers, in fact, are also PPS light stabilizers. Hence, it should be noted that aside fromhigh efficiency in thermal processes, these additives should also have high light resistance [36].

## 4. Conclusions

It was found that the coloring of a PPS reinforced by short glass fibers at photochemical or thermal aging is partially reversible and can be changed by the effect of visible light. During photoaging, cycles of coloring–partial discoloration can be repeated many times, and thus the composite shows a kind of photochromism.

Partial discoloration under the effect of visible light is due to the oxidation of polyconjugated structures on the surface of the composite.

Photodegradation only affects a narrow surface layer of the composite with a thickness of 20 to 40 μm, depending on the conditions and the exposure duration.

Coloring during thermal aging is thermally irreversible. The activation energy of the process in the range of 90 °C to 100 °C is 110 kJ/mol. The deviation at higher temperatures from the Arrhenius law is, apparently, due to a change in the molecular mobility during the transition through the glass transition temperature recorded by the DTMA method.

The feature of partial discoloration of the thermally aged samples when they are exposed to visible light is the predominant change in the coordinate b*, which characterizes yellowness, but not lightness L*, which determines the change in color difference for photochemically aged samples.

The color change during thermal decomposition occurs in the layers of the composite located deep inside from the surface, and the color at a depth of 50 to 100 μm does not significantly differ from the color of the surface. However, subsequent radiation by the visible light that causes a significant color change only occurs in a narrow surface layer with a thickness of about 20 μm.

Since photochemical coloring takes place in a thin surface layer, even a relatively long exposure (120 h) of samples 2–4 mm thick does not affect the physicomechanical and electrotechnical characteristics of the composite. The exceptions are the Charpy impact strength (decreased by 21%), however, even that remained at a high enough level that it didnot impose limitations on the scope of the material.

The use of quantitative colorimetry as an additional method of non-destructive testing of the stability of glass-filled composites based on PPS can be advised, however, it must take the partially reversible change of color into account.

## Figures and Tables

**Table 1:** The change in the activation energy, E_α_, with increasing color difference, ΔE, during irradiation of the composite in the SUNTEST device.

**ΔE**	2	3	4	5	6	8	10	12
**E_α_, kJ/mol**	34	39	34	31	25	20	19	16
**ΔE**	16	18	20	21	22	23	24	26
**E_α_, kJ/mol**	18	21	26	29	32	35	36	37

**Table 2:** The physicomechanical and electrical characteristics of the composite after long-term (120 h) irradiation by total light in the Q-SUN device at an intensity of 1000 W/m^2^ and black panel temperature of 50 °C.

Measured Property and Unit	Test Method	Obtained Value	
		Before Irradiation	After Irradiation
Tensile stress at break, MPa	ISO 527-2:2012	185.0 ± 4.8	170.2 ± 5.6
Tensile modulus, MPa	ISO 527-2:2012	16,410 ± 35	16,400 ± 43
Flexural stress at break, MPa	ISO 178:2010	271.0 ± 3.9	262.8 ± 4.2
Flexural modulus, MPa	ISO 179-1:2010	12,930 ± 36	12,950 ± 40
Charpyunnotched impact strength, kJ/m^2^	ISO 179-1:2010	50.0 ± 1.8	39.3 ± 2.1
Volume resistivity, Ω	ICE 93-80	8 × 10^15^ ± 1 × 10^15^	5 × 10^15^ ± 1×10^15^
Surface resistivity, Ω.cm	ICE 93-80	6 × 10^16^ ± 1 × 10^15^	6 × 10^16^ ± 1 × 10^15^
